# Fatal poisoning of Old Polish ducks with *Amanita muscaria*

**DOI:** 10.1186/s12917-026-05461-4

**Published:** 2026-04-11

**Authors:** Dagmara Stępień-Pyśniak, Krzysztof Tutaj, Jowita S. Niczyporuk, Bartosz Sell, Agnieszka Marek, Karolina Piekarska, Katarzyna Ognik

**Affiliations:** 1 Department of Veterinary Prevention and Avian Diseases, Faculty of Veterinary Medicine, University of Life Sciences in Lublin, Lublin, Poland; 2 Department of Biochemistry and Toxicology, Faculty of Animal Sciences and Bioeconomy, University of Life Sciences, Lublin, Poland; 3 Department of Virology and Animal Viral Diseases, National Veterinary Institute – National Research Institute in Pulawy, Pulawy, Poland; 4 Department of Chemical Research of Food and Feed, Toxicological Diagnostics Research Team, National Veterinary Institute – National Research Institute in Pulawy, Pulawy, Poland

**Keywords:** Red fly agaric, Red toadstools, Duck poisoning, Muscimol, Ibotenic acid

## Abstract

**Background:**

*Amanita muscaria* (fly agaric) is a poisonous mushroom containing ibotenic acid (IBA) and muscimol (MUS), two neuroactive alkaloids capable of causing severe or fatal intoxication. While human poisoning is well documented, information on fatal intoxication in birds is limited. This report presents the first documented case of fatal poisoning of Old Polish ducks following ingestion of *A. muscaria*, confirmed by anatomopathological and toxicological analyses.

**Methods:**

Post-mortem specimens, including blood, heart, brain, kidney, liver, lung, pectoral and femoral muscles, and gastrointestinal contents, were subjected to LC–MS/MS analysis for the determination of IBA and MUS. Tissue samples were homogenized, extracted, derivatized, and quantified using multiple reaction monitoring. Mushroom caps and stems collected from the environment were analyzed using the same analytical approach. To exclude alternative toxicological etiologies, liver, muscle, kidney, and gastric contents were screened for rodenticides, pesticides, mycotoxins, and other chemical toxicants by LC–MS/MS. Viral infections were excluded by PCR and RT-PCR assays targeting DNA and RNA viruses commonly affecting waterfowl. In addition, comprehensive bacteriological, mycological, and parasitological examinations were conducted.

**Results:**

Post-mortem examination revealed lamellar mushroom fragments in the glandular stomach and congestion in the caeca and brain. Ibotenic acid (IBA) concentrations across tissues ranged from 4 to 1987 µg/kg, while muscimol (MUS) ranged from 2 to 66 µg/kg. In gastrointestinal contents, IBA and MUS concentrations ranged from 16.2 to 1110.5 µg/g and from 2 to 41.3 µg/g, respectively. Analysis of environmental mushroom material showed higher toxin levels in caps (871.7 µg/g IBA; 197.5 µg/g MUS) than in stems (206.6 µg/g IBA; 15.3 µg/g MUS). Screening of liver, muscle, kidney, and gastric contents excluded the presence of rodenticides, pesticides, mycotoxins, and other chemical toxicants, while PCR/RT-PCR and comprehensive bacteriological, mycological, and parasitological examinations ruled out viral, bacterial, fungal, and parasitic infections.

**Conclusion:**

The presence of IBA and MUS in all organs confirms *A. muscaria* poisoning as the cause of death. This case highlights the toxicological risk of fly agaric ingestion in waterfowl and indicates that meat and tissues from affected birds are unsafe for consumption.

**Supplementary Information:**

The online version contains supplementary material available at 10.1186/s12917-026-05461-4.

## Background

The fly agaric (*Amanita muscaria*) is a mushroom commonly found in broadleaf and coniferous forests, especially in regions with a temperate climate in the Northern Hemisphere. In Poland, it is most commonly found from July to November. A characteristic feature of the fly agaric is its bright red cap covered with white spots or warts. Its fruiting bodies reach up to 20 cm in height, while the caps reach up to 21 cm in diameter [1].

*A. muscaria* is a poisonous mushroom. Its main active substances are ibotenic acid (IBA) and muscimol (MUS) [2]. Toxins cross the blood–brain barrier, most likely through an active transport system, and as neurotransmitter agonists, they exert their effects mainly on the central nervous system (CNS) [3]. The combination of these compounds can cause a wide range of clinical signs, depending on the amount consumed, the relative amounts of toxins in the mushroom, and the patient’s overall physical condition. Symptoms, reported in humans, include intoxication, hallucinations, anxiety, psychomotor agitation, CNS depression, and gastrointestinal disturbances [4, 5].

Mushroom poisoning is a common cause of human hospitalization in summer and autumn [5, 6]. Several cases (including fatal ones) of *A. muscaria* poisoning in dogs have also been reported [7, 8]. However, there is a lack of scientific data on natural poisoning of birds by lamellar fungi such as the red fly agaric.

Poultry and other animals kept in backyard husbandry systems are exposed not only to various pathogens, but also to toxic substances such as plant pesticides, rodenticides, and poisonous plants and fungi [9–13].

Although avian mycoses and mycotoxicoses caused by lower fungi are relatively well characterized, data on *Amanita muscaria* intoxication in birds remain scarce. No studies have documented naturally occurring cases or quantified the tissue distribution of its neurotoxic alkaloids, highlighting a significant gap in understanding the toxicological risks posed to waterfowl. Our study addresses this gap by providing the first analytically confirmed case of *A. muscaria* intoxication in ducks.

Given the above, the aim of this study was to describe a case of *A. muscaria* poisoning in backyard-reared Old Polish ducks. The study also presents a detailed toxicological analysis (including IBA, MUS and other potentially toxic substances) of various tissues collected during anatomopathological examination of all three birds. The concentration of alkaloids was also determined in a fresh fly agaric mushroom growing in the vicinity of the mushrooms eaten by the ducks. In addition, to rule out other causes of bird mortality, diagnostics were performed for bacterial, fungal and parasitic infections and for the detection of genetic material of eight viruses pathogenic to ducks.

## Results

### Post-mortem lesions

**Fig. 1:**
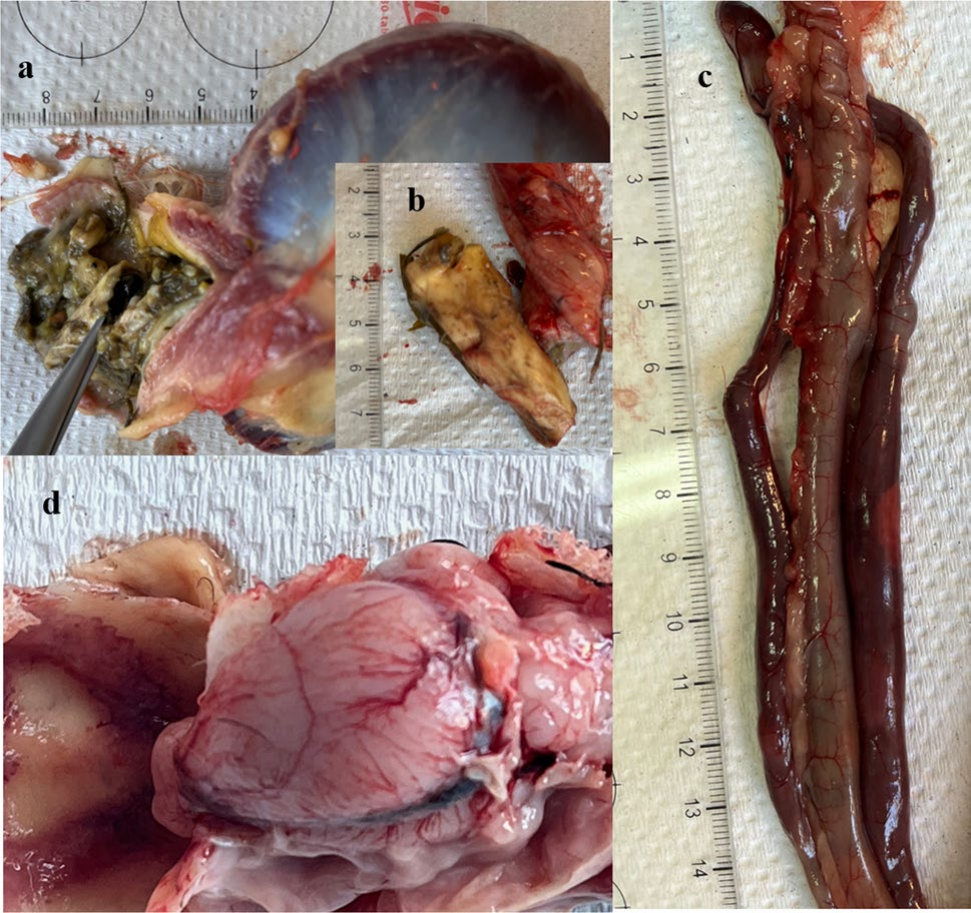
(**a**) Red fly agaric in proventriculus; (**b**) Fragment of *A. muscaria* removed from proventriculus; (**c**) Congestion of the caecum; (**d**) Congestion of the brain

The post-mortem examination of all three drakes revealed fragments of lamellar fungi in the proventriculus (Fig. 1a and b) as well as congestion of the caecum (Fig. 1c) and brain (Fig. 1d).

**Fig. 2:**
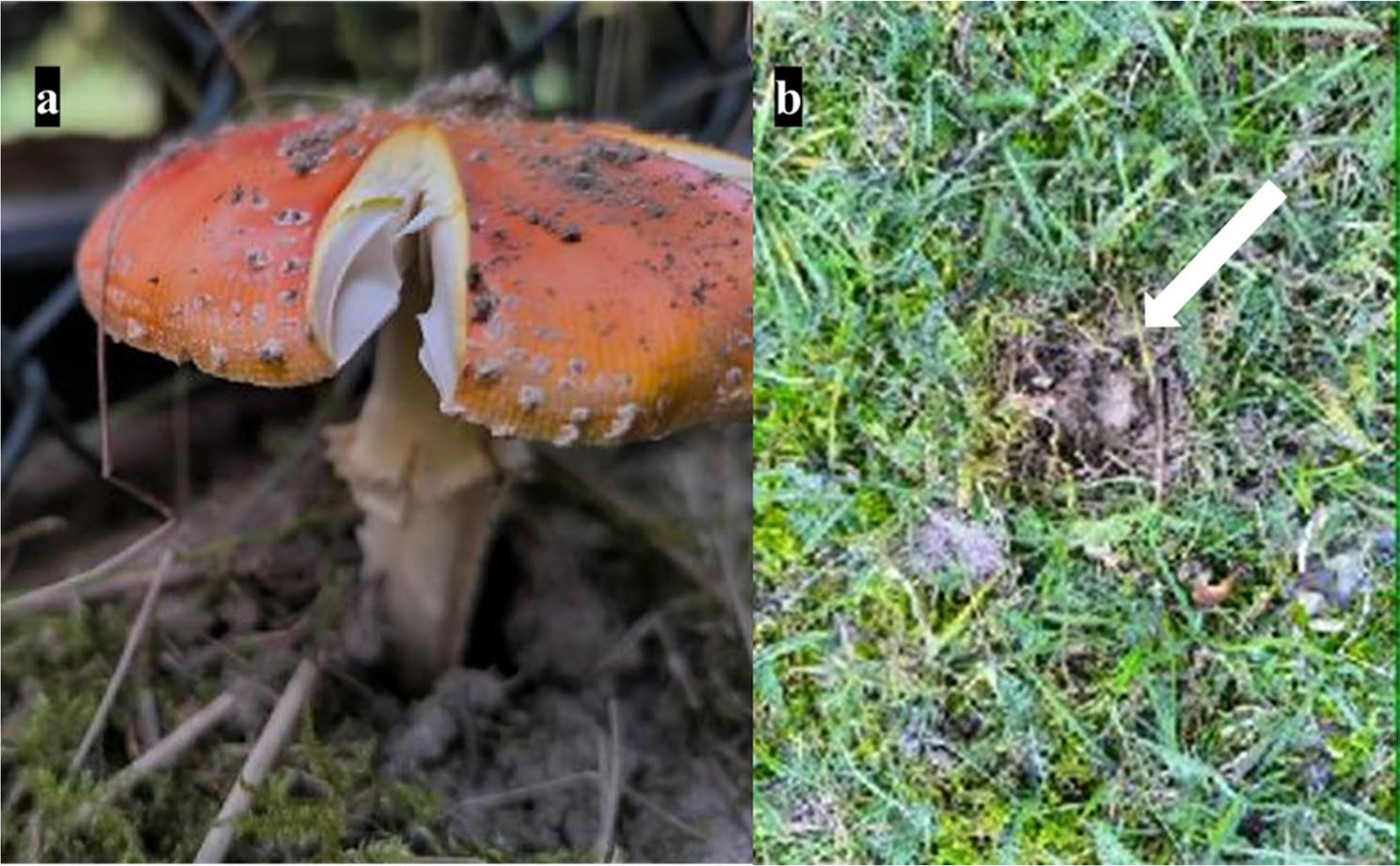
(**a**) Red toadstools growing near those eaten by ducks; (**b**) Location in the soil where a toadstool was eaten by ducks

After performing the anatomopathological examination, the veterinarian informed the owner of the birds of the likely cause of their death. It was then established that there were Caucasian fir trees in the duck enclosure, next to which red fly agaric mushrooms had been growing (Fig. 2a). The owner of the ducks confirmed that the mushrooms were missing and that there were holes in the ground where they had been (Fig. 2b).

### Bacteriological, mycological and parasitological examination

No growth of gram-positive or gram-negative bacteria, mould fungi, or yeasts was found in the material taken from the air sacs, lungs, heart, liver, or brain. The parasitological examination did not reveal the presence of parasites in the intestinal contents.

#### Molecular analysis of viral infections

No genetic material of goose parvovirus (GPV), Muscovy duck parvovirus (MDPV), goose circovirus (GoCV), goose haemorrhagic polyomavirus (GHPV), duck enteritis herpesvirus (DVE), duck hepatitis virus type I (DHV), reovirus (ARV), and adenoviruses (FAdV) was found in the organ samples (liver, spleen, heart, kidneys, stomach, or duodenum) (Figures S1-S8).

### Toxicological analysis

Depending on the internal organ examined for toxicological testing, the concentration of IBA ranged from 4 µg/kg to 1987 µg/kg, while MUS ranged from 2 µg/kg to 66 µg/kg (Table 1).

**Table 1:** Content of ibotenic acid (IBA) and muscimol (MUS) in the organs of drakes

Duck Number	Organ/Tissue	IBA(µg/kg)	MUS(µg/kg)
Drake 1	Brain^*^	nt	nt
	Kidney	961	14
	Liver	141	5
	Lung	196	32
	Heart	71	17
	Femoral muscles	75	32
	Pectoral muscles	4	10
	Heart blood	37	11
Drake 2	Brain	7	9
	Kidney	1806	23
	Liver	49	2
	Lung	131	30
	Heart	72	15
	Femoral muscles	5	19
	Pectoral muscles	6	22
	Heart blood	70	14
Drake 3	Brain	90	13
	Kidney	1987	30
	Liver	124	4
	Lung	840.5	66
	Heart	277.5	44
	Femoral muscles	102.5	24
	Pectoral muscles	119.5	36
	Heart blood	351.5	60

^*^nt – not tested; the duck was killed by decapitation, and its head was not supplied for examination

In the contents of the crops, stomachs, and intestines of the three male ducks, the IBA and MUS content ranged from 16.2 to 1110.5 µg/g and 2 to 41.3 µg/g, respectively (Table 2).

**Table 2:** Concentrations of ibotenic acid (IBA) and muscimol (MUS) in the digestive tract contents and fresh red fly agaric

Origin	Sample	IBA [µg/g]	MUS [µg/g]
Drake 1	Crop contents	27.6	16.5
	Stomach contents	90.1	17.3
	Intestinal contents	16.3	1.2
Drake 2	Crop contents	28.9	3.6
	Stomach contents	43.7	10.9
	Intestinal contents	1100.5	41.3
Drake 3	Crop contents	16.2	2.0
	Stomach contents	21.1	3.9
	Intestinal contents	82.8	2.5
Mushroom	Cap	871.7	197.5
	Stem	206.6	15.3

The ibotenic acid content in the cap and stem of fresh red fly agaric mushrooms was 871.7 µg/g and 206.6 µg/g, respectively, while the muscimol content was 197.5 µg/g and 15.3 µg/g (Table 2).

In addition to targeted IBA and MUS determination, an ancillary qualitative LC–ESI–MS/MS screening was performed to exclude other toxicants as alternative causes of death. The screening was used for presence/absence assessment only; no quantitative conclusions were derived from this dataset. No compounds from the applied multiclass toxicological screening panel (barbiturates, fungicides, coccidiostats, molluscicides, mycotoxins, neonicotinoids, pesticides, carbamate pesticides, and rodenticides) were detected above the screening detection limits defined for the method (typically SDL ≤ 10 µg/kg) in samples of livers, muscles, kidneys, and stomach contents.

## Discussion

Every year, many countries report cases of red fly agaric poisoning in humans as a result of accidental or intentional consumption of these mushrooms. People intentionally consume fresh or dried red fly agaric mushrooms to induce altered states of consciousness, for medicinal purposes, for spiritual needs, or to enhance mental performance [14]. As a result, the toxicological profile and clinical course of *A. muscaria* intoxication are relatively well described in human medicine.

Although *Amanita muscaria* is classified as a poisonous mushroom, it does not cause adverse health effects in some wild animals, such as squirrels [15]. In the case of domestic animals (e.g. dogs and Old Polish ducks – the case described here), ingestion of *A. muscaria* results in clinical signs of poisoning similar to those observed in humans, sometimes even leading to death. According to information published on the website of the North American Mycological Association, dogs willingly consume fly agaric due to its fish-like smell (‘Mushroom Poisonings in Dogs and Cats’ – North American Mycological Association). It is difficult to determine whether the same characteristic contributed to the consumption of *A. muscaria* by the Old Polish drakes.

The owner of the ducks examined in the study has kept Old Polish ducks for many years due to the high quality of their meat. According to the owner, this was the first time the ducks had showed any interest in and eaten fly agaric mushrooms, despite the fact that these mushrooms had grown in the same place in the enclosure every year. The Old Polish ducks were regularly fed *ad libitum*. Despite this, in October 2024 the birds became interested in *A. muscaria*. This unusual behavior may have been influenced by environmental factors. In recent years, Poland has experienced markedly higher autumn temperatures and reduced precipitation compared to long-term climatic norms, including in October 2024, when the poisoning occurred [16]. Such conditions may influence fungal growth, alkaloid concentration, and animal foraging behavior.

The toxic effects of *A. muscaria* are primarily mediated by two neuroactive alkaloids: ibotenic acid and muscimol. After ingestion, these compounds are rapidly absorbed from the gastrointestinal tract and cross the blood–brain barrier, most likely via active transport mechanisms [1]. Ibotenic acid acts predominantly as an excitatory agonist at N-methyl-D-aspartate (NMDA) and metabotropic glutamate receptors [17], whereas muscimol is a potent γ-aminobutyric acid (GABA A​) receptor agonist, resulting in CNS depression [18]. Importantly, ibotenic acid undergoes decarboxylation in the acidic environment of the gastrointestinal tract, being converted into muscimol. This metabolic conversion explains the characteristic transition between the two clinical phases of poisoning.

Clinically, *A. muscaria* intoxication follows a biphasic course. The initial excitatory phase, dominated by IBA, is characterized by hyperactivity, agitation, incoordination, tremors, mydriasis, hallucinations, and gastrointestinal disturbances [19, 20]. This phase is followed by a depressive phase, dominated by MUS, manifested by CNS depression, lethargy, ataxia, hypothermia, muscle hypotonia, and, in severe cases, coma or respiratory failure [5, 20]. The duration and severity of each phase depend on the dose ingested, the extent of IBA-to-MUS conversion, and species-specific sensitivity. In the case described here, the owner of the birds observed rapid deterioration of the drakes’ condition with severe intoxication progressing from an excitatory phase to profound CNS depression. However, it was not possible to precisely determine the time that had elapsed between the ingestion of fly agaric mushrooms and the appearance of specific clinical signs.

In humans, gastrointestinal signs such as vomiting, diarrhea, and abdominal pain may appear within 15–30 min after ingestion. Neurological symptoms—including weakness, dizziness, disorientation, mydriasis, tinnitus, and visual or auditory hallucinations—typically develop within 30 min to two hours [19, 20]. Subsequently, fatigue and somnolence progress to deep sleep, which is often considered the terminal stage of uncomplicated poisoning [5]. In more severe cases, pronounced psychomotor agitation, muscle rigidity, seizures, hyperthermia, coma, or respiratory failure may occur [20]. Although precise temporal relationships could not be established in the ducks, the observed clinical course appears broadly comparable, suggesting similar toxicodynamic mechanisms in birds and humans.

During the anatomopathological examination, fragments of the lamellar mushroom were found in the glandular stomach of all three drakes. Toxicological analysis of stomach contents demonstrated IBA concentrations ranging from 21.1 to 90.1 µg/g and MUS concentrations from 3.9 to 17.3 µg/g. Fresh *A. muscaria* mushrooms are known to be particularly toxic due to their high ibotenic acid content. A fly agaric fruiting body weighing 50–70 g (fresh weight) can contain up to 70 mg of ibotenic acid and approximately 6 mg of muscimol [1]. Based on toxicological studies of fresh fly agaric mushrooms growing in the vicinity of the mushrooms eaten by the ducks, we showed that their cap and stem contained comparable IBA and MUS concentrations to those reported by Carboué and Lopez [1].

Based on these values, the reported consumption of three mushrooms, and an average drake body weight of approximately 5 kg, the estimated exposure was ~ 23.4 mg/kg for IBA and ~ 4.9 mg/kg for MUS. These doses are consistent with severe intoxication and likely sufficient to explain the fatal outcome. For comparison, psychoactive effects in humans may occur after ingestion of a single mushroom [21], whereas the estimated lethal dose corresponds to approximately 15 fresh caps [20]. Experimental studies in mice report LD_50_ values of 38 mg/kg for ibotenic acid and 22 mg/kg for muscimol [22], supporting the plausibility of lethal toxicity at the estimated avian doses. In addition, De Carolis et al. [23] demonstrated that muscimol administered intraperitoneally at 0.5–1 mg/kg affects the electroencephalogram of cats and rabbits.

Toxicological analysis revealed the presence of IBA and MUS in each of the internal organs of the Old Polish ducks, which shows that their meat and other tissues would not have been suitable for consumption, as even boiling do not degrade these toxins [24]. The IBA and MUS content was 4–119.5 in the pectoral and muscles and 10–36 µg/kg in the femoral muscles. However, the highest concentrations of these alkaloids were found in the kidneys (ibotenic acid 961–1987 µg/kg) and lungs (muscimol 30–66 µg/kg).

In the case of poisoning of a dog with red fly agaric, Rossmeisl et al. [7] noted that ibotenic acid concentrations determined by High-Performance Liquid Chromatography were 0.706 and 0.239 mg/ml in the urine and serum, respectively. In addition, muscimol was present in the serum and urine at concentrations of 0.083 and 4.814 mg/ml, respectively. In contrast, in blood samples taken from the hearts of the three drakes, the IBA and MUS concentrations were in the ranges of 37–351.5 and 11–60 µg/kg, respectively.

## Conclusion

This study reports the first analytically confirmed case of fatal *Amanita muscaria* intoxication in Old Polish ducks kept under backyard husbandry conditions. A comprehensive diagnostic approach identified ibotenic acid and muscimol as the cause of death and excluded infectious diseases and alternative toxicological etiologies.

The presence of both alkaloids in all examined tissues, including edible organs, indicates systemic distribution of *A. muscaria* toxins in ducks and suggests that meat from affected birds may pose a food safety risk due to the thermal stability of these compounds. High toxin concentrations detected in gastrointestinal contents and in environmental mushroom samples support ingestion of fresh fly agaric fruiting bodies as the source of exposure.

The main limitations of this report include the small number of affected animals, the retrospective nature of the investigation, and the lack of precise information regarding the timing and dose of exposure. Nevertheless, this case highlights *A. muscaria* as a previously underrecognized toxicological hazard in waterfowl and underscores the importance of routine monitoring and removal of poisonous mushrooms from backyard poultry environments.

## Materials and methods

### Case history

Three dead Old Polish ducks aged approximately 6 months, including one killed humanely by decapitation to end its suffering, were delivered to the Department of Veterinary Prevention and Avian Diseases of the Faculty of Veterinary Medicine, University of Life Sciences in Lublin, Poland. The ducks’ owner reported that the drakes had shown no signs of illness in the morning and had behaved as usual. In the afternoon, the owner noticed that the birds had difficulty moving and their heads were shaking. The owner compared the drakes’ clinical signs to the behaviour of someone having consumed large amounts of alcohol. In addition, the birds had tried to defend themselves, attacking the owner when she approached. Shortly afterwards, the drakes stopped moving and lay on their sternum with their heads stretched forward. They did not respond to external stimuli. A few hours later, two of them died, and the third, which was near death, was euthanized by the owner to end its suffering. The drakes had been fed oats, steamed potatoes and commercial feed mix *ad libitum* and had access to a limited outdoor run.

### Anatomopathological, bacteriological, mycological, and parasitological examination

An anatomopathological examination of the dead birds was performed. Material for bacteriological testing was collected from the air sacs, lungs, heart, liver, and brain. It was cultured on MacConkey Agar (Biomaxima. Poland), Columbia Agar with 5% sheep blood (Biomaxima, Poland), and CNA Staph/Strep Selective Agar (Biomaxima, Poland) and incubated at 37 °C under aerobic and microaerophilic conditions for up to 48 h. Material for mycological testing was collected from the air sacs and lungs and then cultured on Sabouraud agar (Biomaxima, Poland) and incubated at 37 °C under aerobic conditions for 7 days. The contents of the small intestines and caecum were collected for parasitological examination. Testing was carried out using the McMaster centrifugal technique with a saturated NaCl and glucose solution as the flotation liquid and by the decantation method [25].

### Detection of viral infection

To detect possible infection with duck viruses, samples were taken from the liver, spleen, heart, kidneys, stomach, and duodenum, from which 20% homogenates were prepared. For this purpose, sections of each organ were suspended in Eagle’s fluid (MEM, Sigma Aldrich, USA) enriched with a 1% addition of a mixture of antibiotics (Antibiotic-Antimycotic, Gibco, Scotland). To release viral particles from the cells, the homogenates were subjected to three cycles of freezing and thawing. The materials were then centrifuged (4 °C, 3000 × g for 5 min), and the supernatants were collected for isolation of total cellular DNA and RNA. Genetic material was isolated from homogenates of internal organs collected from the dead birds. A standard procedure was applied according to the manufacturer’s instructions for the commercial IndiMag Pathogen Kit (INDCIAL, Germany). The resulting eluate was stored at temperatures below − 20 °C until further analyses.

Tests were performed for the presence of genetic material of GPV, MDPV, GoCV, GHPV, DVE, DHV, ARV, and FAdV. To detect the genetic material of the viruses, polymerase chain reaction (PCR) was used for DNA viruses and reverse transcription followed by polymerase chain reaction (RT-PCR) for RNA viruses. Oligonucleotide sequences based on conserved regions of the genomes of the viral strains were used for this purpose (Table S1). Primers were synthesized by Genomed (Poland). Working primers at concentrations of 10 µM were used in the reactions. The reactions were carried out in a gradient thermocycler (Biometra, Germany) in a final reaction mixture volume of 25 µl.

The time and temperature conditions used in PCR for the detection of genetic material of DNA viruses are presented in Table S2. The time and temperature conditions used in RT-PCR for the detection of genetic material of RNA viruses are shown in Table S3.

PCR products were separated on a 2% agarose gel with GelRed™ Nucleic Acid Gel Stain (Biotium, USA). MassRuler Ladder LR (80–1031 bp) (Thermo Fisher Scientific, USA) was used as a molecular size marker. A 10 µl volume of each PCR product was loaded into the gel wells. Electrophoresis was carried out at 120 V for 50 min in TEA buffer (50×, AppliChem PanReac). The separation results were visualized under UV light (Bio-Rad, USA). Agarose gels were documented and photographed under UV illumination using a gel documentation system (VWR Genosmart, Germany).

The sensitivity of the reaction was confirmed by real-time PCR using the same primers. The following Ct results were observed in the tested samples: GPV Ct = 21, MDPV Ct = 24, GHPV Ct = 16, GoCV Ct = 18, DHV Ct = 24, DVE Ct = 22, ARV Ct = 21.

### Determination of ibotenic acid and muscimol in mushrooms and digestive tract contents by liquid chromatography–tandem mass spectrometry (LC-MS/MS)

The procedure is an adaptation of the method routinely used in screening for IBA and MUS by the Institute of Forensic Research in Kraków. A 20 mg sample was weighted into 2 ml bead mill tubes and homogenized using a soft tissue programme (Bead Mill Max Homogenizer, VWR International LLC, Randor, USA). Prior to homogenization, 10 µl of internal standard (1 µg ml^− 1^ of L-tyrosine-13C9,15 N, Merck), 2 µl of formic acid, and 180 µl of methanol/water solution were added. The mixtures were extracted ultrasonically for 15 min, shaken for one hour, left overnight at 6 °C, and centrifuged the next day at 14,000 × g for 5 min. Extracts were transferred to a glass vial with insert for LC-MS/MS analysis. The method validation protocol and data are included in the supplementary material (Table S4). Chromatographic separation was carried out on a Kinetex Biphenyl (100 mm × 3 mm, 2.6 μm) column (Phenomenex, Torrance, USA). The mobile phase consisted of (A) water containing 0.1% v/v of formic acid (initially 95%) and (B) acetonitrile containing 0.1% v/v of formic acid (5%); 0.4 mL/min flow. Gradient programme: 0.0–1.5 min 5% B, 1.5–7.0 min 5–95% B, 7.0–9.0 min hold 95% B, 9.0–9.5 min 95–5% B, 9.5–13.0 min 5% B. The injection volume was 1 µl. Electrospray ionization in the positive ion mode (ESI+) was used for detection. MS interface parameters were set as follows: curtain gas (CUR) = 35 psi, collision gas (CAD) = 12 (high), electrospray voltage = 5500 V, nebulizing gas (GS1) = 70 psi, auxiliary gas and temperature (GS2) = 60 psi and 600 °C. Tandem mass spectrometry MS/MS was used for quantification. MRM transitions and settings were optimized by infusing individual derivatized standard solutions with a syringe pump. The parameters of all molecules monitored by the MRM method, i.e. precursor (Q1), product ions (Q2), collision energy (CE), collision cell exit potential (CXE), and retention times, are listed in Table S5.

### Determination of ibotenic acid and muscimol in post-mortem specimens by LC-MS/MS

The method described by Xu et al. [26], with modifications, was applied for ibotenic acid and muscimol analysis in blood from the heart and in the heart muscle, brain, kidney, liver, lung, and pectoral and femoral muscles. A total of 200 mg of tissue was mixed with 200 µl of water and 300 mg of ceramic beads (1.4 mm diameter) in 2 ml bead mill tubes and homogenized using a soft tissue programme (Bead Mill Max Homogenizer, VWR International LLC, Randor, USA). Prior to homogenization, 10 µl of internal standard (1 µg ml^− 1^ of L-tyrosine-13C9,15 N, Merck) and 5 µl of formic acid were added. Then, protein precipitation was performed in triplicate. A 200 µl volume of acetonitrile was added to 100 µl of blood or 100 mg of homogenate. Next, the extracts were centrifuged at 14,000 × g for 2 min, and 156 µl of supernatant was taken for derivatization with dansyl chloride (30 min. incubation at 60 °C). After the reaction, the test tube was cooled down and neutralized using 50 µl of 37% hydrochloric acid and mixed with 1 ml of dichloromethane. The extracts were centrifuged at 14,000 × g for 2 min, and the organic layer was collected into a new tube and evaporated at 30 °C under a stream of nitrogen. The dry residue was reconstituted in 100 µL of a water–acetonitrile mixture (1:1, v/v) and centrifuged at 14,000×g. The supernatant was transferred to a glass vial with insert for LC-MS/MS analysis. The validation protocol and data are presented in Table S4.

Ibotenic acid and muscimol concentrations were determined using a high performance liquid chromatograph (Exion LC AD, AB Sciex, Framingham, MA, USA) coupled with a mass spectrometer (QTRAP 6500+, AB Sciex, Framingham, MA, USA). Chromatographic separation was carried out on a Kinetex Luna Omega Polar C18 100 Å (150 mm × 3 mm, 3.0 μm) column (Phenomenex, Torrance, CA, USA) at column temperature 30 °C and flow rate of 0.4 ml/min. The mobile phase consisted of (A) water containing 10 mM formic acid and 6 mM ammonium formate and (B) acetonitrile. The mobile phase started with 40% B, followed by the following gradient programme: 0.0–0.5 min 40% B, 0.5–7.0 min 40–90% B, 7.0–9.0 min hold 90% B, 9.0–9.5 min 90–40% B, 9.5–13.0 min 40% B. The injection volume was 1 µl. Electrospray ionization in the positive ion mode (ESI+) was used for detection. MS interface parameters were set as follows: curtain gas (CUR) = 35 psi, collision gas (CAD) = 12 (high), electrospray voltage = 5500 V, nebulizing gas (GS1) = 70 psi, auxiliary gas, and temperature (GS2) = 60 psi and 600 °C. Tandem mass spectrometry MS/MS was used for quantification. MRM transitions and settings were optimized by infusing individual derivatized standard solutions with a syringe pump. The parameters of all molecules monitored with the MRM method, i.e. precursor (Q1), product ions (Q2), collision energy (CE), collision cell exit potential (CXE), and retention times are listed in Table S6. The LC-MS/MS system was controlled using Analyst 1.7.2 software (AB Sciex, Framingham, MA, USA). SCIEX OS Version 2.1.6.59781 (AB Sciex, Framingham, MA, USA) was used for data processing.

### Determination of other toxic substances by LC-MS/MS

Liver, muscle, kidney and stomach content samples were subjected to toxicological screening to exclude other potentially toxic substances belonging to multiple chemical classes. Analytes were extracted using a QuEChERS extraction and LC–MS/MS conditions followed the previously developed and validated multiclass method by Sell et al. [27].

Homogenized samples (2 ± 0.05 g) were mixed with 5 ml of acetonitrile and 0.5 g of sodium acetate. The mixture was subjected to vigorous vortex mixing (349 × g for 30 s), followed by sonication for 15 min in an ultrasonic bath. After additional vortex mixing (1 min), the samples were centrifuged at 2,930 × g for 20 min at room temperature.

The supernatant was transferred to a centrifuge tube containing dispersive solid-phase extraction (d-SPE) sorbents. The contents were vortex-mixed for 1 min and centrifuged at 2,930 × g for 10 min at room temperature. The supernatant was filtered through 0.2 μm filters at 9,447 × g for 10 min and then transferred to autosampler vials for analysis.

Chromatographic separation and detection were performed using high-performance liquid chromatography (Shimadzu) coupled with triple quadrupole tandem mass spectrometry (LC-MS/MS), utilizing electrospray ionization (ESI) in both positive and negative ionization modes (ABSciex API 5500 Qtrap).

Chromatographic separation was achieved on a Luna C8 column (75 mm × 2.1 mm × 3 μm) connected to a C8 pre-column (4 mm × 2 mm × 3 μm) maintained at 30 °C. The mobile phase consisted of (A) 5% isopropanol in ethanol and (B) 0.5% isopropanol in 0.1% acetic acid in water, delivered at a flow rate of 0.3 mL/min in the gradient programme.

Mass spectrometry parameters were set as follows: capillary voltage ± 4.5 kV (positive and negative modes), desolvation temperature 550 °C, nebulizer gas (N₂) 35 psi, curtain gas (N₂) 30 psi, collision gas (N₂) medium, gas 1 (air) 35 psi, gas 2 (air) 35 psi, and multiplier voltage 2,100 V. The multiple reaction monitoring (MRM) mode was employed for analyte detection and quantification.

Screening was performed for the following chemicals: rodenticides (bromadiolone, brodifacoum, chloralose, chlorophacinone, diphacinone, difenacoum, difethialone, flocoumafen, coumatetralyl, coumachlor, strychnine, and warfarin), carbamate pesticides (aldicarb, dioxacarb, carbaryl, carbofuran, carbofuran-3-hydroxy, carbofuran-3-keto, methiocarb, oxamyl, pirimicarb, propoxur, and bendiocarb), organophosphate pesticides (azinphos-ethyl, azinphos-methyl, chlorfenvinphos, chlorpyrifos, diazinon, dichlorvos, dicrotophos, dimefox, dimethoate, disulfoton, ethion, etrimfos, phoxim, fonofos, formothion, phosalone, malaoxon, malathion, methacrifos, methamidophos, mevinphos, omethoate, pyrazophos, pirimiphos-ethyl, pirimiphos-methyl, profenofos, propetamphos, sulfotep, and triazophos), barbiturates (pentobarbital), fungicides (cyproconazole and carboxin), coccidiostats (lasalocid, maduramicin, monensin, narasin, salinomycin, and semduramicin), molluscicides (metaldehyde), mycotoxins (aflatoxins B1, B2, G1, and G2; ochratoxin A; T-2 toxin; HT-2 toxin; sterigmatocystin; zearalenone; deoxynivalenol), and neonicotinoids (imidacloprid). Information on the LC‑ESI–MS/MS transitions monitored, statistical analysis, and validation parameters (linearity, reproducibility, repeatability, selectivity, recoveries, matrix effects, robustness, screening detection limits and limits of quantification) was described in detail in our previously published article by Sell et al. [27].

## Supplementary Information

Supplementary Material 1: Table S1. Oligonucleotide sequences used to detect viral genetic material in the organs of Old Polish ducks.

Supplementary Material 2: Table S2. Time and temperature conditions used in PCR for the detection of genetic material of DNA viruses.

Supplementary Material 3: Table S3. Time and temperature conditions used in RT-PCR for the detection of genetic material of RNA viruses.

Supplementary Material 4: Table S4. Parameters of the LC-MS method and validation protocol.

Supplementary Material 5: Table S5. Parameters of IBA, MUS and IS molecules monitored by the MRM method.

Supplementary Material 6: Table S6. Parameters of dansylated molecules monitored by the MRM method.

Supplementary Material 7:Fig. S1. Agarose gel electrophoresis of PCR products for the detection of goose parvovirus (GPV). Lane M: M-MassRuler Ladder Mix DNA Ladder (80–10000 bp). (K-) - negative control; (K+) - positive control (Goose parvovirus H strain isolated from the commercial Palmivax vaccine); Lanes 1–6: liver, spleen, lungs, heart, kidneys, intestines samples, respectively. No specific GPV amplicons were detected in any of the examined organ samples, while the positive control showed the expected PCR product.

Supplementary Material 8:Fig. S2. Agarose gel electrophoresis of PCR products for the detection of mullard duck parvovirus (MDPV). Lane M: M-MassRuler Ladder Mix DNA Ladder (80–10000 bp). NC: negative control; PC: positive control (FM strain - Vilmos Palya, CEVA-Phylaxia, Ceva Sante Animale, Budapest, Hungary); Lanes 1–6: liver, spleen, lungs, heart, kidneys, intestines samples, respectively. No specific MDPV amplicons were detected in any of the examined organ samples, while the positive control showed the expected PCR product.

Supplementary Material 9:Fig. S3. Agarose gel electrophoresis of PCR products for the detection of goose circovirus (GoCV) Lane M: M-MassRuler Low Range DNA Ladder (80–1031 bp). NC: negative control; PC: positive control (39/14 strain - GenBank accession number MH138278); Lanes 1–6: liver, spleen, lungs, heart, kidneys, intestines samples, respectively. No specific GoCV amplicons were detected in any of the examined organ samples, while the positive control showed the expected PCR product.

Supplementary Material 10:Fig. S4. Agarose gel electrophoresis of PCR products for the detection of goose hemorrhagic polyomavirus (GHPV) Lane M: M-MassRuler Low Range DNA Ladder (80–1031 bp). NC: negative control; PC: positive control (50/15 strain - GenBank accession number MG869737); Lanes 1–6: liver, spleen, lungs, heart, kidneys, intestines samples, respectively. No specific GHPV amplicons were detected in any of the examined organ samples, while the positive control showed the expected PCR product (397 bp).

Supplementary Material 11:Fig. S5. Agarose gel electrophoresis of PCR products for the detection of duck enteritis virus (DVE) Lane M: M-MassRuler Low Range DNA Ladder (80–1031 bp). NC: negative control; PC: positive control (1227 strain of Department of Poultry Viral Diseases, NVRI, Pulawy, Poland); Lanes 1–6: liver, spleen, lungs, heart, kidneys, intestines samples, respectively. No specific DVE amplicons were detected in any of the examined organ samples, while the positive control showed the expected PCR product.

Supplementary Material 12: Fig. S6. Agarose gel electrophoresis of PCR products for the detection of duck hepatis virus (DHV) Lane M: M-MassRuler Low Range DNA Ladder (80–1031 bp). NC: negative control; PC: positive control (16/08 strain of Department of Poultry Viral Diseases, NVRI, Pulawy, Poland); Lanes 1–6: liver, spleen, lungs, heart, kidneys, intestines samples, respectively. No specific DHV amplicons were detected in any of the examined organ samples, while the positive control showed the expected PCR product.

Supplementary Material 13:Fig. S7. Agarose gel electrophoresis of PCR products for the detection of reovirus (ARV) Lane M: M-MassRuler Low Range DNA Ladder (80–1031 bp). NC: negative control; PC: positive control (Reovirus S1133 strain isolated from the commercial vaccine); Lanes 1–6: liver, spleen, lungs, heart, kidneys, intestines samples, respectively. No specific ARV amplicons were detected in any of the examined organ samples, while the positive control showed the expected PCR product.

Supplementary Material 14:Fig S8. Agarose gel electrophoresis of PCR products for the detection of adenoviruses (FAdV). Lane M: M-MassRuler Ladder Mix DNA Ladder (80–10000 bp). NC: negative control; PC: positive control (FAdV-1/A Charles River, US); Lanes 1–6: liver, spleen, lungs, heart, kidneys, intestines samples, respectively. No specific FAdV amplicons were detected in any of the examined organ samples, while the positive control showed the expected PCR product.

## Data Availability

The data that support the findings of this study are available from the corresponding author on request.

## References

[1] Carboué Q, Lopez M. *Amanita muscaria*: ecology, chemistry, myths. Encyclopedia. 2021;1:905–914.

[2] Okhovat A, Cruces W, Docampo-Palacios ML, Ray KP, Ramirez GA. Psychoactive isoxazoles, muscimol, and isoxazole derivatives from the *Amanita* (Agaricomycetes) species: review of new trends in synthesis, dosage, and biological properties. *Int J Med Mushrooms*. 2023;25:1–10. 10.1615/IntJMedMushrooms.2023049458 37824402

[3] Grochowska J, Grygoruk N, Weiner M. Harmfulmess and potential health benefits of the fly agaric (*Amanita muscaria*): a literature review. Health Probl Civiliz. 2025;19:95–106.

[4] Vendramin A, Brvar M. *Amanita muscaria* and *Amanita pantherina* poisoning: two syndromes. Toxicon. 2014;90:269–272. 10.1016/j.toxicon.2014.08.067 25173077

[5] Meisel EM, Morgan B, Schwartz M, Kazzi Z, Cetin H, Sahin A. Two cases of severe *Amanita muscaria* poisoning including a fatality. Wilderness Environ Med. 2022;33:412–416. 10.1016/j.wem.2022.06.002 36210279

[6] Satora L, Pach D, Butryn B, Hydzik P, Balicka-Ślusarczyk B. Fly agaric (*Amanita muscaria*) poisoning, case report and review. Toxicon. 2005;45:941–943. 10.1016/j.toxicon.2005.01.005 15904689

[7] Rossmeisl JH Jr, Higgins MA, Blodgett DJ, Ellis M, Jones DE. *Amanita muscaria* toxicosis in two dogs. J Vet Emerg Crit Care. 2006;16:208–214.

[8] Romano MC, Doan HK, Poppenga RH, Filigenzi MS, Bryant UK, Gaskill CL. Fatal Amanita muscaria poisoning in a dog confirmed by PCR identification of mushrooms. J Vet Diagn Invest. 2019;31:485–487. 10.1177/1040638719842897 PMC6838700 30957709

[9] Cortinovis C, Francesca C. Epidemiology of intoxication of domestic animals by plants in Europe. Vet J. 2013;197:163–168. 10.1016/j.tvjl.2013.03.007 23570777

[10] Moshobane MC, Bertero A, Marks C, et al. Plants and mushrooms associated with animal poisoning incidents in South Africa. Vet Rec Open. 2020;7:e000402. 10.1136/vetreco-2020-000402 PMC7678378 33262890

[11] Katagi T, Fujisawa T. Acute toxicity and metabolism of pesticides in birds. J Pestic Sci. 2021;46:305–321. 10.1584/jpestics.D21-028 PMC8640698 34908891

[12] Bonnefous C, Collin A, Guilloteau A, et al. Welfare issues and potential solutions for laying hens in free range and organic production systems: A review based on literature and interviews. Front Vet Sci. 2022;9:952922. 10.3389/fvets.2022.952922 PMC9390482 35990274

[13] Kobylarz D, Paprotny Ł, Wianowska D, Gnatowski M, Jurowski K. 2024. Silent bird poisoning in Poland: reconfirmation of bromadiolone and warfarin as the proximal causes using GC-MS/MS-based methodology for forensic investigations. Pharmaceuticals (Basel). 2024;17:764. 10.3390/ph17060764 PMC11206662 38931431

[14] Pięta-Chrystofiak M, Brohs D. Red fly agaric (*Amanita Muscaria*) consumption among members of internet discussion groups. Stud Paedag Ignatiana. 2023;26:195–221.

[15] Suetsugu K, Gomi K. Squirrel consuming “poisonous” mushrooms. Front Ecol Environ. 2021;19:556–556.

[16] Marosz M, Biernacik B, Kusek K, Wasielewska K, Kitowski M. Characteristics of selected climate elements in Poland in October 2024. Institute of Meteorology and Water Management. National Research Institut. .Assessed November 6, 2025.

[17] Manoguerra AS. Mushrooms, ibotenic acid. In: Wexler P, editors. Encyclopedia of toxicology. 2nd ed., Elsevier Inc., 2005.

[18] Puschner B. Mushroom toxins. In: Gupta RC, editor. Veterinary Toxicology. Academic Press, 2007.

[19] Marciniak B., Ferenc T., Kusowska J., Ciećwierz J., Kowalczyk E. 2010. Zatrucia wybranymi grzybami o działaniu neurotypowym i halucynogennym (In Polish). Med Pr, 61, 583–595. 21341527

[20] Mikaszewska-Sokolewicz MA, Pankowska S, Janiak M, Pruszczyk P, Łazowski T, Jankowski K. Coma in the course of severe poisoning after consumption of red fly agaric (*Amanita muscaria*). Acta Biochim Pol. 2016;63:181–182. 10.18388/abp.2015_1170 26828668

[21] Voynova M., Shkondrov A., Kondeva-Burdina M, Krasteva I. Toxicological and pharmacological profile of *Amanita muscaria* (L.) Lam. – a new rising opportunity for biomedicine. Pharmacia. 2020;67:317–323.

[22] Leas EC, Satybaldiyeva N, Kepner W, et al. Need for a public health response to the unregulated sales of *Amanita muscaria* mushrooms. Am J Prev Med. 2024;67,458–463. 10.1016/j.amepre.2024.05.006 PMC11832274 38864780

[23] De Carolis A, Lipparini F, Longo V. Neuropharmacological investigations on muscimol, a psychotropic drug extracted from *Amanita muscaria*. Psychopharmacologia. 1969;15:186–195. 10.1007/BF00411168 5389124

[24] Tsunoda K, Inoue N, Aoyagi Y, Sugahara T. Change in ibotenic acid and muscimol contents in *Amanita muscaria* during drying, storing or cooking. *J Food Hyg Soc Japan (Shokuhin Eiseigaku Zasshi)*. 1993;34(2):153–160.

[25] Permin A, Hansen JW. Epidemiology, diagnosis and control of poultry parasites. Food and Agriculture Organization of the United Nations. (FAO animal health manual; no. 4), 1998.

[26] Xu XM, Zhang JS, Huang BF, Han JL, Chen Q. Determination of ibotenic acid and muscimol in plasma by liquid chromatography-triple quadrupole mass spectrometry with bimolecular dansylation. J Chromatogr B Analyt Technol Biomed Life Sci. 2020;1146: 122–128. 10.1016/j.jchromb.2020.122128 32361630

[27] Sell B, Sniegocki T, Zmudzki J, Posyniak A. Development of an analytical procedure for the determination of multiclass compounds for forensic veterinary toxicology. J Anal Toxicol. 2018;42:1–9. 10.1093/jat/bkx093 29194519

