# Supplementary material for: Fatal poisoning of Old Polish ducks with Amanita muscaria
Source: BMC Vet Res. 2026 Apr 11;22:301. doi: 10.1186/s12917-026-05461-4 (PMC13195819; doi:10.1186/s12917-026-05461-4)
Supplement: Supplementary file 1 — Supplementary Material 1: Table S1. Oligonucleotide sequences used to detect viral genetic material in the organs of Old Polish ducks. [file 12917_2026_5461_MOESM1_ESM.docx]

**Table S1.** Oligonucleotide sequences used to detect viral genetic material in the organs of Old Polish ducks

| **Virus** | **Primers** | **Sequences 5’-3’** | **Amplification product size** | **References** |
| --- | --- | --- | --- | --- |
| Goose haemorrhagic polyomavirus (GHPV) | GHPV-F | ACCCGTGCTTCCATTCACAA | 397 bp | Corrand et al., 2011 |
|  | GHPV-R | CTGCTCCCCAAACCTGTCAA |  |  |
| Goose circovirus (GoCV) | ORFC1-F | GGAAGGGGTTAAATGCGGGA | 308 bp | Chen et al., 2003 |
|  | ORFC1-R | ACGATACAGACGACGAAGGC |  |  |
| Goose parvovirus (GPV) | VP3F | GTGCCGATGGAGTGGGTAAT | 1604 bp | Woźniakowski et al., 2009 |
|  | VP3R | GCGCCAGGAAGTGCTTTAT |  |  |
| Reovirus (ARV) | 967F | CCCACTTTCCATTCCTTTCA | 967 bp | Woźniakowski et al., 2014  Kant et al., 2003 |
|  | 967R | GCCATCCTAGCTGGAGAGAC |  |  |
| Reovirus (ARV)  nested PCR | sigmaNSF | CCGAGTGGCCCTATTGACTA | 508 bp | Zhang et al., 2016 |
|  | sigmaNSR | CAGCGACCACTTAGATGCAA |  |  |
| Duck enteritis herpesvirus (DVE) | DVE F | TGCGACCTTAATTTCGATGG | 300 bp | Hanses et al., 2000 |
|  | DVE R | CCATACTTGGATGGGTCAGC |  |  |
| Duck hepatitis picornavirus (DHV-1) | DHV1F | GCCAATGTATATGATCTGCCA | 742 bp | Mangisa et al., 2004 |
|  | DHV1R | GTGGCTGCTCGAACTCTACG |  |  |
| Muscovy duck parvovirus (MDPV1) | MDPV1 | GGAGAGAAATGGCAGTGG | 1204 bp | Mangisa et al., 2004 |
|  | MDPV2 | GCTGTTGTTGTGTTTTGT |  |  |
| Adenovirus (FAdV) | FAdV-F | AATGTCACNACCGARAAGGC | 830 bp | Niczyporuk et al., 2016 |
|  | FAdV-R | CBGCBTRCATGTACTGGTA |  |  |

**References**

1. Chen CL, Chang PC, Lee MS, Shien JH, Ou SJ, Shien HK. Nucleotide sequences of goose circovirus isolated in Tawian. Avian Pathol. 2003;32:165-171.
2. Corrand L, Gelfi J, Albaric O, Etievant M, Pingret JL. Guerin pathological and epidemiological significance of goose haemorrhagic polyomavirus infection in ducks. Avian Pathol. 2011;40:355-360.
3. Hansen WR, Nashold SW, Docherty DE, Brown SE, Knudson DL. Diagnosis of duck plague in waterfowl by polymerase chain reaction. Avian Dis. 2000;44:266-74.
4. Hansen WR, Nashold SW, Docherty DE, Brown SE, Knudson DL. Diagnosis of duck plague in waterfowl by polymerase chain reaction. Avian Dis. 2000;44:266-74.
5. Kant A., balk F., born L., van RoozelaarD., Heijmans J., Gielkens A. Classification of Dutch and German avian reoviruses by sequencjing the sigma C protein. Vet Res. 2003;34:203-212.
6. Mangisa NP, Smuts HE, Kramvis A, Linley CW, Skelton M, Tucker TJ, De La M Hall P, Kahn D, Jilbert AR, Kew MC. Molecular characterization of duck hepatitis B virus isolates from South African ducks. Virus Genes. 2004;28:179-86.
7. Niczyporuk J.S.N., Phylogenetic and geographic analysis of fowl adenovirus field strains isolated from poultry in Poland. Arch Virol. 2016;161:33–42.
8. Woźniakowski G., Kozdruń W., Samorek-Salamonowicz E. Genetic variance of Derzsy's disease strains isolated in Poland. J Mol Genet Med . 2009 Nov 30;3(2):210–216. doi: 10.4172/1747-0862.1000037
9. Woźniakowski G., Samorek-Salamonowicz E., Gaweł A. Occurrence of reovirus infection in Muscovy ducks (*Carina moschata*) in south western Poland. Pol J Vet Sci. 2014;17:299-305.
10. Zhang Y, Liu M., Shuidong O., Hu Q.L., Guo D.C., Chen H.Y., Han Z.: Detection and identification of avian , duck, and goose reoviruses by RT-PCR: goose and duck reoviruses are part of the same genogroup in the genus Orthoreovirus. Arch Virol. 2006;151:1525-1538.
