# Supplementary material for: Fatal poisoning of Old Polish ducks with Amanita muscaria
Source: BMC Vet Res. 2026 Apr 11;22:301. doi: 10.1186/s12917-026-05461-4 (PMC13195819; doi:10.1186/s12917-026-05461-4)
Supplement: Supplementary file 2 — Supplementary Material 2: Table S2. Time and temperature conditions used in PCR for the detection of genetic material of DNA viruses. [file 12917_2026_5461_MOESM2_ESM.docx]

**Table S2.** Time and temperature conditions used in PCR for the detection of genetic material of DNA viruses

| **Virus** | **Initial denaturation** | **Denaturation** | **Primer binding** | **Elongation** | **Number of cycles** | **Final elongation** |
| --- | --- | --- | --- | --- | --- | --- |
| GPV | 95℃/5 min | 94℃/15 s | 60℃/20 s | 72℃/45 s | 40 | 72℃/10 min |
| MDPV | 95℃/5 min | 94℃/15 s | 60℃/20 s | 72℃/45 s | 40 | 72℃/10 min |
| GHPV | 95℃/5 min | 94℃/15 s | 55℃/20 s | 72℃/45 s | 35 | 72℃/10 min |
| GoCV | 95℃/5 min | 94℃/15 s | 54℃/20 s | 72℃/45 s | 35 | 72℃/10 min |
| DVE | 95℃/5 min | 94℃/15 s | 54,5℃/1 min | 72℃/1 min | 35 | 72℃/10 min |
| REO nested | 95℃/5 min | 94℃/1 min | 60℃/1 min | 72℃/1 min | 35 | 72℃/10 min |
| FAdV | 95°C/5 min | 94°C/45 s | 55°C/1 min | 72°C/2 min | 35 | 72°C/10 min |

goose parvovirus (GPV), Muscovy duck parvovirus (MDPV), goose circovirus (GoCV), goose haemorrhagic polyomavirus (GHPV), duck enteritis herpesvirus (DVE), duck hepatitis virus type I (DHV), as well as reovirus (ARV) and adenoviruses (FAdV)
