# Supplementary material for: Fatal poisoning of Old Polish ducks with Amanita muscaria
Source: BMC Vet Res. 2026 Apr 11;22:301. doi: 10.1186/s12917-026-05461-4 (PMC13195819; doi:10.1186/s12917-026-05461-4)
Supplement: Supplementary file 3 — Supplementary Material 3: Table S3. Time and temperature conditions used in RT-PCR for the detection of genetic material of RNA viruses. [file 12917_2026_5461_MOESM3_ESM.docx]

**Table S3**. Time and temperature conditions used in RT-PCR for the detection of genetic material of RNA viruses

| **Virus** | **Reverse transcription** | **Initial denaturation** | **Denaturation** | **Primer binding** | **Elongation** | **Number of cycles** | **Final elongation** |
| --- | --- | --- | --- | --- | --- | --- | --- |
| DHV | 50℃/30 min | 95℃/15 min | 94℃/45 s | 52℃/1 min | 72℃/1 min | 40 | 72℃/10 min |
| REO | 50℃/30 min | 95℃/15 min | 94℃/45 s | 57℃/1 min | 72℃/1 min | 40 | 72℃/10 min |
