# Supplementary material for: Fatal poisoning of Old Polish ducks with Amanita muscaria
Source: BMC Vet Res. 2026 Apr 11;22:301. doi: 10.1186/s12917-026-05461-4 (PMC13195819; doi:10.1186/s12917-026-05461-4)
Supplement: Supplementary file 4 — Supplementary Material 4: Table S4. Parameters of the LC-MS method and validation protocol. [file 12917_2026_5461_MOESM4_ESM.docx]

**Table S4.** Parameters of the LC-MS method.

| **Analyte** | **Matrix** | **LOD** | **LOQ** | **Linearity range** | **R^2^** | **Recovery (%)** | **Accuracy**  **(%)** | **Precision (%)** | **Matrix effect (%)** |
| --- | --- | --- | --- | --- | --- | --- | --- | --- | --- |
| IBA | blood | 1 µg/kg | 5 µg/kg | 5 – 500 µg/kg | 0,994 | 94 | 47 | 8,38 | 31 |
| IBA | liver | 3 µg/kg | 5 µg/kg | 5 – 500 µg/kg | 0.976 | 140 | 102 | 15,52 | 58 |
| IBA | mushroom | 0.01 µg/g | 0.05 µg/g | 0.05 – 100 µg/g | 0.992 | 95 | 109 | 1.99 | 44 |
| MUS | blood | 0.05 µg/kg | 0.1 µg/kg | 0.1 – 500 µg/kg | 0.997 | 94 | 98 | 1,90 | 67 |
| MUS | liver | 0.5 µg/kg | 1 µg/kg | 1 – 500 µg/kg | 0.986 | 122 | 99 | 12,99 | 72 |
| MUS | mushroom | 0.005 µg/g | 0.01 µg/g | 0.01-100 µg/g | 0.997 | 106 | 104 | 0,76 | 67 |

**Validation protocol**

The LC–MS method was validated through a comprehensive evaluation of linearity, limits of detection (LOD), limits of quantification (LOQ), matrix effects, recovery, accuracy, and precision. Matrix matched calibration curves for IBA and MUS with concentrations from 0.01 to 100 µg/g (for mushrooms and digestive tract contents analysis) and within the range 0.1 – 500 µg/kg (for organ/tissue analysis) were constructed by plotting the peak area ratio of the target compound to internal standard versus the concentration of IBA or MUS. *Pleurotus ostreatus* was selected as a blank mushroom matrix for the construction of calibration curves for MUS and IBA, as it exhibited no interfering peaks in the retention time region of the analytes. In addition to *Pleurotus ostreatus*, the following edible mushrooms were evaluated: *Agaricus bisporus*, *Lentinula edodes*, *Pholiota nameko* and *Pleurotus eryngii*. All mushrooms were obtained from a single producer (Tomasz Grela, Dąbrówka, Poland), originated from the same cultivation batch, and were produced under certified ecological conditions. The selection of *Pleurotus ostreatus* as a blank matrix is consistent with the findings of Tsujikawa and collaborators, who indicated that this species may be used as a blank mushroom matrix [1, 2]. Linearity was evaluated by least-squares regression to obtain the slope, intercept, coefficient of determination (R²), and overall linearity performance. LOD and LOQ were estimated statistically from the standard deviation of replicate measurements at the lowest concentration level and the slope of the calibration curve (LOD ≥ 3×SD/slope; LOQ ≥ 10×SD/slope). Accuracy (%) was calculated with the following formula: determined value/true value × 100. Precision (%) was defined as the ratio of the standard deviation to the mean (n=10). For recovery experiment (%, n = 6) the known amounts of MUS and IBO were spiked into 20 mg of blank mushroom or to 100 mg of acetonitrile precipitated blank blood/liver. Than the response of extracted analyte in spiked blank matrix was compared with the response of analyte spiked after the extraction of blank matrix. The matrix effect (%bias, n = 6) was determined by comparing the response of analyte spiked after the extraction of blank matrix with the response of analyte in neat solution.

**References**

1. Tsujikawa K, Kuwayama K, Miyaguchi H, Kanamori T, Iwata Y, Inoue H, Yoshida T, Kishi T. Determination of muscimol and ibotenic acid in Amanita mushrooms by high-performance liquid chromatography and liquid chromatography-tandem mass spectrometry. J Chromatogr B Analyt Technol Biomed Life Sci. 2007, 852(1-2):430-5.
2. Tsujikawa K, Mohri H, Kuwayama K, Miyaguchi H, Iwata Y, Gohda A, Fukushima S, Inoue H, Kishi T. Analysis of hallucinogenic constituents in *Amanita mushrooms* circulated in Japan. Forensic Sci Int. 2006, 164(2-3):172-8.
