# Supplementary figures and images for: Fatal poisoning of Old Polish ducks with Amanita muscaria
Source: BMC Vet Res. 2026 Apr 11;22:301. doi: 10.1186/s12917-026-05461-4 (PMC13195819; doi:10.1186/s12917-026-05461-4)

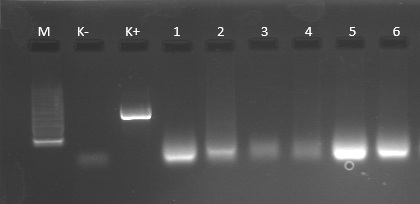

Supplement: Supplementary file 7 — Supplementary Material 7:Fig. S1. Agarose gel electrophoresis of PCR products for the detection of goose parvovirus (GPV). Lane M: M-MassRuler Ladder Mix DNA Ladder (80–10000 bp). (K-) - negative control; (K+) - positive control (Goose parvovirus H strain isolated from the commercial Palmivax vaccine); Lanes 1–6: liver, spleen, lungs, heart, kidneys, intestines samples, respectively. No specific GPV amplicons were detected in any of the examined organ samples, while the positive control showed the expected PCR product. [file 12917_2026_5461_MOESM7_ESM.png]

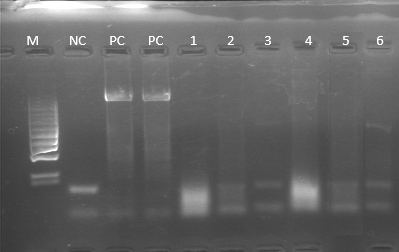

Supplement: Supplementary file 8 — Supplementary Material 8:Fig. S2. Agarose gel electrophoresis of PCR products for the detection of mullard duck parvovirus (MDPV). Lane M: M-MassRuler Ladder Mix DNA Ladder (80–10000 bp). NC: negative control; PC: positive control (FM strain - Vilmos Palya, CEVA-Phylaxia, Ceva Sante Animale, Budapest, Hungary); Lanes 1–6: liver, spleen, lungs, heart, kidneys, intestines samples, respectively. No specific MDPV amplicons were detected in any of the examined organ samples, while the positive control showed the expected PCR product. [file 12917_2026_5461_MOESM8_ESM.png]

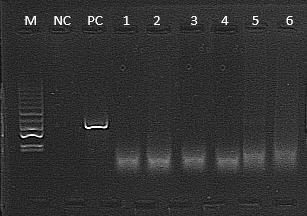

Supplement: Supplementary file 9 — Supplementary Material 9:Fig. S3. Agarose gel electrophoresis of PCR products for the detection of goose circovirus (GoCV) Lane M: M-MassRuler Low Range DNA Ladder (80–1031 bp). NC: negative control; PC: positive control (39/14 strain - GenBank accession number MH138278); Lanes 1–6: liver, spleen, lungs, heart, kidneys, intestines samples, respectively. No specific GoCV amplicons were detected in any of the examined organ samples, while the positive control showed the expected PCR product. [file 12917_2026_5461_MOESM9_ESM.png]

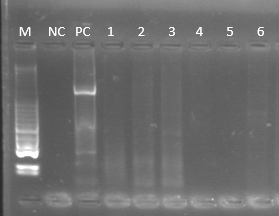

Supplement: Supplementary file 10 — Supplementary Material 10:Fig. S4. Agarose gel electrophoresis of PCR products for the detection of goose hemorrhagic polyomavirus (GHPV) Lane M: M-MassRuler Low Range DNA Ladder (80–1031 bp). NC: negative control; PC: positive control (50/15 strain - GenBank accession number MG869737); Lanes 1–6: liver, spleen, lungs, heart, kidneys, intestines samples, respectively. No specific GHPV amplicons were detected in any of the examined organ samples, while the positive control showed the expected PCR product (397 bp). [file 12917_2026_5461_MOESM10_ESM.png]

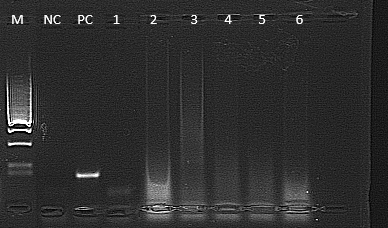

Supplement: Supplementary file 11 — Supplementary Material 11:Fig. S5. Agarose gel electrophoresis of PCR products for the detection of duck enteritis virus (DVE) Lane M: M-MassRuler Low Range DNA Ladder (80–1031 bp). NC: negative control; PC: positive control (1227 strain of Department of Poultry Viral Diseases, NVRI, Pulawy, Poland); Lanes 1–6: liver, spleen, lungs, heart, kidneys, intestines samples, respectively. No specific DVE amplicons were detected in any of the examined organ samples, while the positive control showed the expected PCR product. [file 12917_2026_5461_MOESM11_ESM.png]

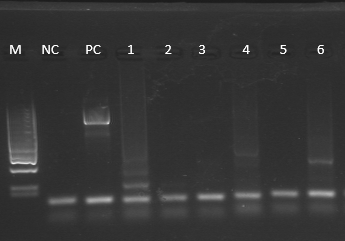

Supplement: Supplementary file 12 — Supplementary Material 12: Fig. S6. Agarose gel electrophoresis of PCR products for the detection of duck hepatis virus (DHV) Lane M: M-MassRuler Low Range DNA Ladder (80–1031 bp). NC: negative control; PC: positive control (16/08 strain of Department of Poultry Viral Diseases, NVRI, Pulawy, Poland); Lanes 1–6: liver, spleen, lungs, heart, kidneys, intestines samples, respectively. No specific DHV amplicons were detected in any of the examined organ samples, while the positive control showed the expected PCR product. [file 12917_2026_5461_MOESM12_ESM.tif]

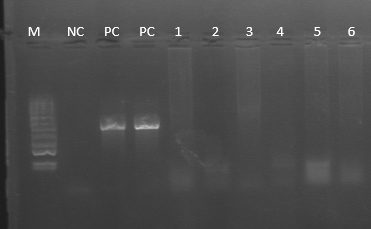

Supplement: Supplementary file 13 — Supplementary Material 13:Fig. S7. Agarose gel electrophoresis of PCR products for the detection of reovirus (ARV) Lane M: M-MassRuler Low Range DNA Ladder (80–1031 bp). NC: negative control; PC: positive control (Reovirus S1133 strain isolated from the commercial vaccine); Lanes 1–6: liver, spleen, lungs, heart, kidneys, intestines samples, respectively. No specific ARV amplicons were detected in any of the examined organ samples, while the positive control showed the expected PCR product. [file 12917_2026_5461_MOESM13_ESM.png]

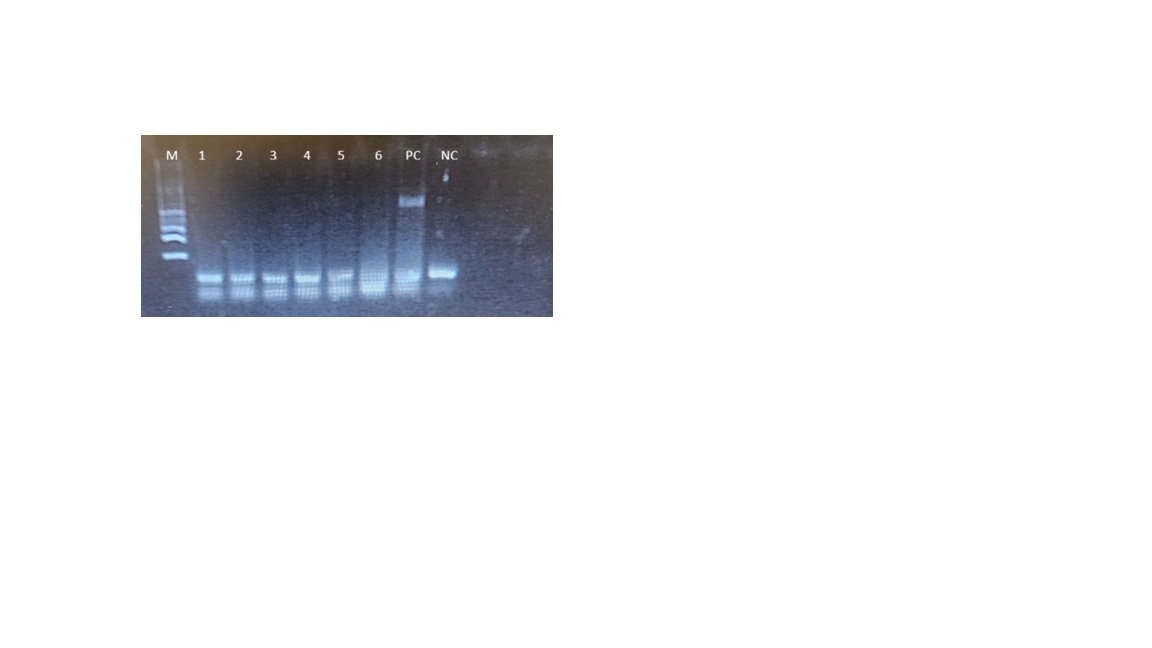

Supplement: Supplementary file 14 — Supplementary Material 14:Fig S8. Agarose gel electrophoresis of PCR products for the detection of adenoviruses (FAdV). Lane M: M-MassRuler Ladder Mix DNA Ladder (80–10000 bp). NC: negative control; PC: positive control (FAdV-1/A Charles River, US); Lanes 1–6: liver, spleen, lungs, heart, kidneys, intestines samples, respectively. No specific FAdV amplicons were detected in any of the examined organ samples, while the positive control showed the expected PCR product. [file 12917_2026_5461_MOESM14_ESM.jpg]
